# Patient’s Desire and Real Availability Concerning Supportive Measures Accompanying Radical Prostatectomy: Differences between Certified Prostate Cancer Centers and Non-Certified Centers Based on Patient-Reported Outcomes within the Cross-Sectional Study Improve

**DOI:** 10.3390/cancers15102830

**Published:** 2023-05-19

**Authors:** Ingmar Wolff, Martin Burchardt, Julia Peter, Christian Thomas, Danijel Sikic, Christian Fiebig, Sören Promnitz, Bernd Hoschke, Maximilian Burger, Marco J. Schnabel, Christian Gilfrich, Niklas Löbig, Nina N. Harke, Florian A. Distler, Matthias May

**Affiliations:** 1 Department of Urology, University Medicine Greifswald, 17475 Greifswald, Germany; 2 Department of Urology, St. Elisabeth Hospital Straubing, 94315 Straubing, Germany; 3 Department of Urology, University Hospital Carl Gustav Carus, Technische Universität Dresden, 01307 Dresden, Germany; 4 Department of Urology and Pediatric Urology, University Hospital Erlangen, Friedrich-Alexander-University Erlangen-Nuremberg, 91054 Erlangen, Germany; 5 Department of Urology, Klinikum Frankfurt (Oder), 15236 Frankfort (Oder), Germany; 6 Department of Urology and Pediatric Urology, Carl-Thiem-Klinikum Cottbus, 03048 Cottbus, Germany; 7 Department of Urology, Caritas-St. Josef Medical Center, University of Regensburg, 93053 Regensburg, Germany; 8 Department of Urology, University Hospital Ulm, 89081 Ulm, Germany; 9 Department of Urology and Urologic Oncology, Hanover Medical School, 30625 Hanover, Germany; 10 Department of Urology, Paracelsus Medical University Nuremberg, 90419 Nuremberg, Germany

**Keywords:** prostate cancer, radical prostatectomy, perioperative supportive measures, certified prostate cancer centers, patient-reported outcome, survey

## Abstract

**Simple Summary:**

This German multicenter study investigated the importance of different supportive measures offered to patients with prostate cancer who undergo surgery (radical prostatectomy). A number of these supportive measures are required during the certification of a urologic hospital as prostate cancer center. However, a broad scientific basis evaluating these measures from the patient’s perspective is still lacking. In this study, patients were asked to rate the relevance of several supportive measures and to estimate the effective availability of these different supportive measures at their urologic clinic about 15 months after surgery. Our study highlights that only six of fifteen different supportive measures were rated as very relevant by patients. None of these six supportive measures were offered more intensively at the certified clinics compared to the non-certified clinics according to the patients. Our study helps to identify those supportive measures with the highest subjective impact on patients in this setting.

**Abstract:**

Certification as a prostate cancer center requires the offer of several supportive measures to patients undergoing radical prostatectomy (RP). However, it remains unclear how patients estimate the relevance of these measures and whether the availability of these measures differs between certified prostate cancer centers (CERTs) and non-certified centers (NCERTs). In 20 German urologic centers, a survey comprising questions on the relevance of 15 supportive measures was sent to 1000 patients at a median of 15 months after RP. Additionally, patients were asked to rate the availability of these measures using a four-item Likert scale. The aim of this study was to compare these ratings between CERTs and NCERTs. The response rate was 75.0%. In total, 480 patients underwent surgery in CERTs, and 270 in NCERTs. Patients rated 6/15 supportive measures as very relevant: preoperative medical counselling concerning treatment options, a preoperative briefing answering last questions, preoperative pelvic floor exercises (PFEs), postoperative PFEs, postoperative social support, and postoperative rehabilitation addressing physical fitness recovery. These ratings showed no significant difference between CERTs and NCERTs (*p* = 0.133–0.676). In addition, 4/9 of the remaining criteria were rated as more detailed by patients in CERTs. IMPROVE represents the first study worldwide to evaluate a patient-reported assessment of the supportive measures accompanying RP. Pertinent offers vary marginally between CERTs and NCERTs.

## 1. Introduction

Radical prostatectomy represents one of the proposed standard treatment options for localized prostate cancer [1,2,3]. Besides oncological and functional outcome parameters, such as cancer-specific and overall survival parameters, urinary continence rates and the extent of preservation of erectile function, patient-reported outcome measures have been observed with increasing interest in the treatment–evaluation of patients undergoing prostate cancer treatment in recent years [4,5,6,7,8,9,10].

In an effort to improve treatment quality, the German Cancer Society (DKG) started to certify cancer centers following predefined criteria, including a number of qualitative and quantitative minimum requirements. Initially, this certification process was established for breast cancer in 2003 and colorectal cancer in 2006 [11]. In the following years, this program was expanded to a large number of cancer entities, including prostate cancer [11,12]. Consequently, such cancer centers were certified by the DKG in Germany and Austria, in addition to German-speaking areas in Italy, Switzerland and Luxemburg [13].

Among the issues required for the certification of a cancer center, several supportive measures have to be offered to patients undergoing radical prostatectomy for prostate cancer [12,14]. However, it remains unclear how patients estimate the relevance of these supportive measures and whether the availability of these measures differs between certified prostate cancer centers (CERTs) and non-certified centers (NCERTs). Therefore, the aim of the “Importance of various supportive measures in the context of radical prostatectomy from the patient’s perspective study (IMPROVE study)” was to assess the relevance of different perioperative supportive measures from the patient’s perspective and to compare the patient-reported effective availability of these supportive measures between CERTs and NCERTs within a large cohort in a contemporary multicenter setting.

## 2. Materials and Methods

From April to June 2021, a survey comprising questions concerning the availability of several supportive measures, in addition to personal, perioperative, and functional criteria, was sent to 1000 patients at a median of 15 months (interquartile range, IQR: 11–21) after radical prostatectomy in 20 German urologic centers (50 patients per center). The following 15 supportive measures were included in the survey:

- preoperative medical counselling concerning the best treatment option for the given patient
- preoperative briefing answering last questions given by a member of the medical team/the surgeon
- preoperative pelvic floor exercises
- preoperative genetic counselling
- preoperative psycho-oncological support
- preoperative integration in a support group of prostate cancer patients
- postoperative pelvic floor exercises
- sufficient postoperative social support
- postoperative rehabilitation addressing recovery of physical fitness
- postoperative genetic counselling
- postoperative nutrition consultation
- postoperative psycho-oncological support
- postoperative access to a pain service
- postoperative integration into a support group of prostate cancer patients
- postoperative counselling regarding therapy options for possible erectile dysfunction.

Using a four-item Likert scale, patients were asked to evaluate the relevance of each supportive measure. The following possible answers were provided: “very important”, “rather important”, “rather irrelevant”, and “irrelevant”. A supportive measure was considered very relevant in the patients’ perspective if it had been rated as “very important” by >50% of patients and “rather important” or “very important” by >80% of patients. 

Patients were also asked to rate the effective availability of every given supportive measure in their center. For this purpose, another four-item Likert scale was used offering the following possible answers: “in great detail”, “in detail”, “little detailed”, and “was not offered”. 

One reminder was sent to those patients who did not respond to the mailed survey.

Within the survey, questions extracted from a validated questionnaire were used to evaluate urinary stress incontinence [15,16,17]. Furthermore, patients were asked to report the way the clinical decision making regarding the surgical approach was performed: passive decision (by physician alone), consensual decision making by patient and physician together, or active decision making (by patient alone).

The final questionnaire response rate was 75.0%, resulting in a study cohort of 750 patients. Data from the survey were merged with details provided by the participating centers: the surgical approach, the prostate-specific antigen (PSA) level prior to surgery, the date of surgery, details concerning nerve preservation, International Society of Urological Pathology (ISUP) groups, the TNM stage, the surgical margin status, and complications during hospital stay recorded according to the Clavien–Dindo Classification (CDC) [18,19]. Finally, the center’s level of care (university vs. non-university) and the center’s mean caseload per year between 2018 and 2020 were included in the analysis. 

Continuous variables were documented as medians with interquartile ranges. The pertinent characteristics of patients undergoing radical prostatectomy in CERTs and NCERTs were compared using the Mann–Whitney test for non-normally distributed variables, and categorical variables were compared using Fisher’s exact test and Pearson’s Chi-squared test.

Finally, the ratings of the subjectively perceived availability of those supportive measures that were rated very relevant by patients were merged within a cumulative score, resulting in a comprehensive presentation comparing the medians of these scores between CERTs and NCERTs.

All statistical analyses were performed using IBM SPSS Statistics^®^ V29 (Armonk, NY, USA). The reported *p* values were two-sided, and the statistical significance level was set at *p* < 0.05.

The study was conducted in accordance with the Declaration of Helsinki. Approval was obtained by the Leading Ethics Committee of Medizinische Hochschule Brandenburg (ethical approval E-01-20200805, date of approval: 17 August 2020). The IMPROVE study was registered in the German register of clinical studies (DRKS-ID: DRKS00023765, date of registration: 22 December 2020).

## 3. Results

The response rate was 75.0% (750/1000). In total, 480 patients underwent surgery in CERTs, and 270 in NCERTs. All participating 8 university centers and 4/12 non-university centers were CERTs. CERTs had a statistically significant higher annual caseload, with a median of 125 vs. 29 radical prostatectomies performed per year during the time period of 2018–2020 compared to NCERTs (*p* < 0.001). The time interval between radical prostatectomy and the survey assessing patients’ ratings of the different supportive measurements differed significantly between CERTs and NCERTs, with a median of 14 (IQR: 11–21) vs. 17 (IQR: 12–21) months (*p* < 0.001).

There were no essential differences in most of the demographic criteria between both groups except for age, which was significantly lower in CERTs (median: 67 vs. 69 years, *p* < 0.001). Concerning oncological criteria, a higher percentage of ISUP groups 3–5 (45.4% vs. 34.8%, *p* = 0.005) and a higher rate of positive surgical margins (26.7% vs. 20.0%, *p* 0.042) were observed in CERTs. The surgical approach also varied significantly between both groups, with a higher proportion of robot-assisted procedures in CERTs vs. NCERTs (68.3% vs. 14.8%, *p* < 0.001) (Table 1).

**Figure 1:**
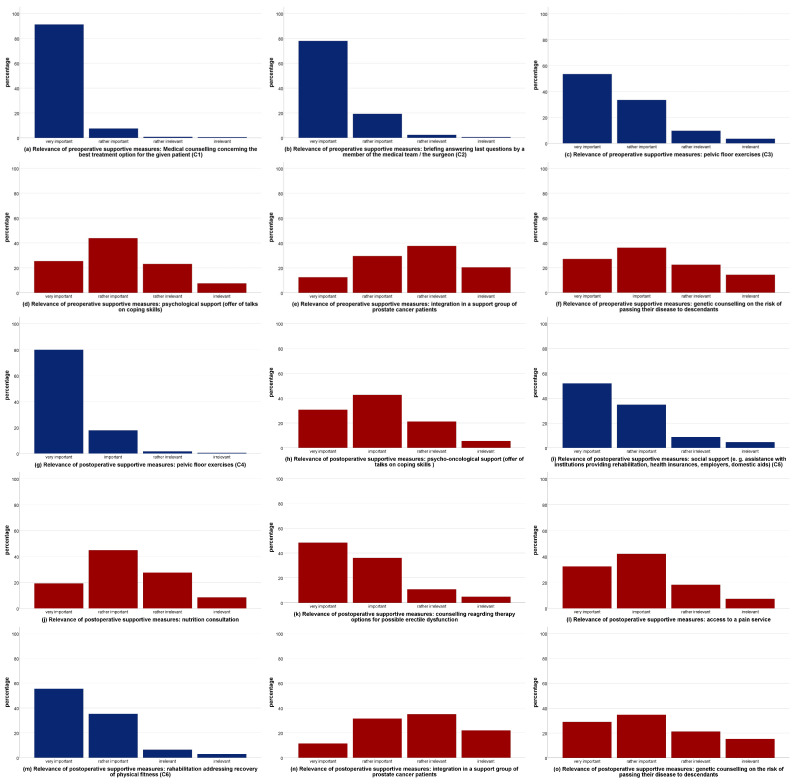
(**a**–**o**): Relevance of supportive perioperative measures. Supportive measures rated as very relevant by patients (C1–C6) were displayed in blue color, all others were displayed in brown color. Legend: CERT, certified prostate cancer center; NCERT, non-certified center.

**Figure 2:**
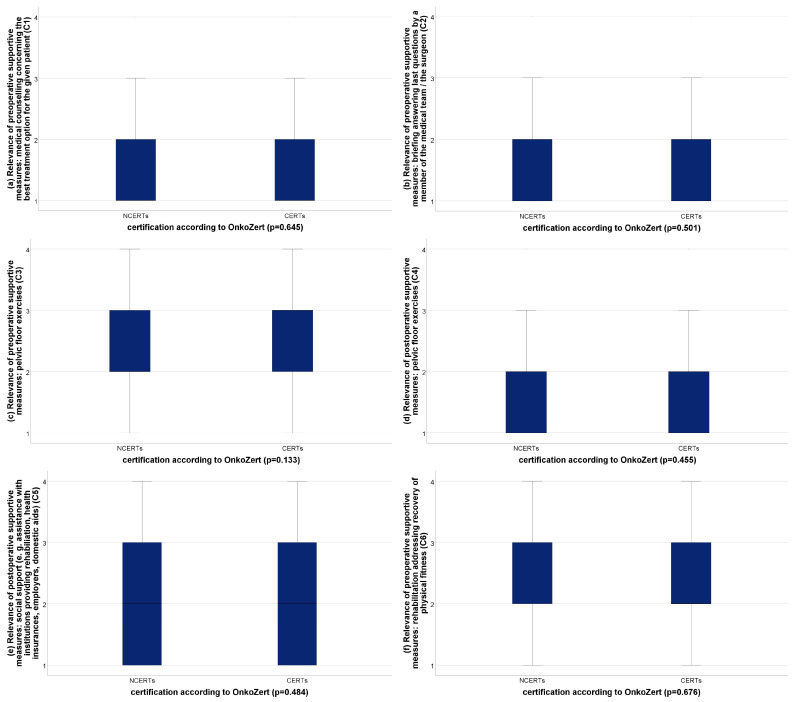
(**a**–**f**): Effective availability of supportive perioperative measures. Legend: CERT, certified prostate cancer center; NCERT, non-certified center.

The patients rated six of the fifteen supportive measures as very relevant: preoperative medical counselling concerning the best treatment option (criterion 1, C1), a preoperative briefing answering last questions given by a member of the medical team/the surgeon (C2), preoperative pelvic floor exercises (C3), postoperative pelvic floor exercises (C4), sufficient postoperative social support (C5), and postoperative rehabilitation addressing the recovery of physical fitness (C6) (Figure 1a–o, displayed in blue color, all others displayed in brown color). None of these ratings showed a significant difference concerning the effective availability estimated by the patients between CERTs and NCERTs (*p* = 0.133–0.676) (Figure 2a–f).

Nine (three preoperative and six postoperative) supportive measures were not rated as very relevant by patients according to the predefined criteria (Figure 1a–o). Of these, four supportive measures were rated as more detailed by patients in CERTs vs. NCERTs: pre- and postoperative genetic counselling (*p* < 0.001 and *p* = 0.003, respectively), postoperative nutrition consultation (*p* < 0.001), and postoperative psycho-oncological support (*p* = 0.013).

**Figure 3:**
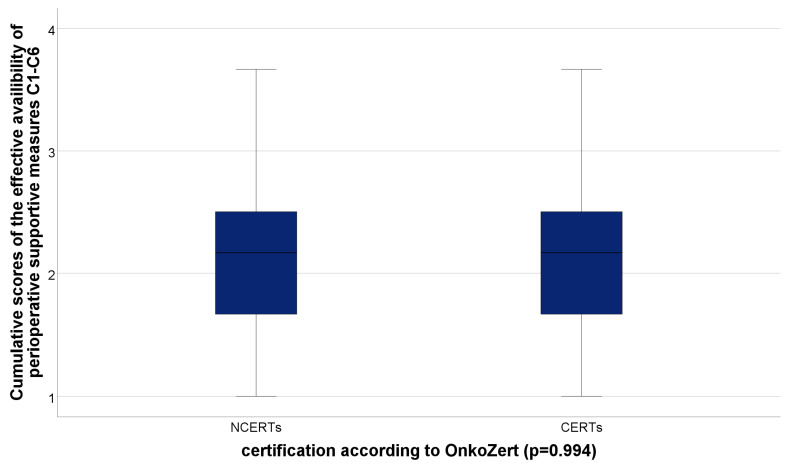
Cumulative scores of the effective availability of those supportive perioperative measures rated as very relevant by patients (C1–C6). Legend: CERT, certified prostate cancer center; NCERT, non-certified center.

Merging the ratings of the subjectively perceived availability of those supportive measures that were rated very relevant by patients (C1–C6) within a cumulative score resulted in a median of exactly 2.17 (IQR: 2.16–2.50) for the effective availability ratings of both groups, CERTs and NCERTs, with no statistically significant difference (*p* = 0.994) (Figure 3).

## 4. Discussion

Despite a number of competing therapeutic approaches, radical prostatectomy remains one of the standard therapy options for localized prostate cancer [1,2,3]. However, in a previous analysis of the IMPROVE study, we found that more than a third of patients who underwent prostatectomy showed critical decision regret at a median of 15 months after surgery [20]. Meissner et al. were even able to show in their longitudinal study that patient’s decision regret continues to increase over longer periods after radical prostatectomy [21]. In these two studies, preoperative shared decision making regarding the choice of therapy was found to be the strongest independent factor influencing the patient’s decision regret, with shared decision making reducing critical decision regret by 38% and 45%, respectively [20,21]. Thus, shared decision making between the patient and his treating physician is an important perioperative criterion that strongly correlates with patients’ long-term acceptance of the surgical procedure. In addition to the intended impact of radical prostatectomy on the oncological outcomes, this procedure clearly has a functional component, resulting in stress urinary incontinence (in varying degrees) and erectile dysfunction in some of the surgically treated patients, leading to a significant impact on the quality of life [22,23,24]. For this reason, perioperative pelvic floor muscle training should be offered to patients by urologic centers, and therapeutic counselling should be offered to patients with postoperative erectile dysfunction problems in an outpatient setting [25,26,27]. Additionally, multidisciplinary patient counselling, given by a surgeon and a radiotherapist together prior to the decision on the best treatment option, may further help to reduce patients’ decision regret in some cases. In this context, the results of a study by Hamdy et al. may be presented to the patients in an effort to assist in decision making, as it provides outcome data derived from a prospective comparison of radical prostatectomy, radiotherapy and active monitoring in patients with localized prostate cancer [28].

Discussions about the necessary number of cases per year for high-quality radical prostatectomy and the demand for the firm establishment of supportive perioperative measures that go hand in hand with a higher treatment acceptance by the patient have led to the certification of prostate cancer centers by the German Cancer Society since 2008 [11,12]. In addition to the strict implementation of case volume requirements and functional rehabilitation support for patients after radical prostatectomy, successful certification as a prostate cancer center in Germany requires a whole range of other supportive measures. In addition, the implementation of these supportive measures is thoroughly controlled within the repeating recertification process of each prostate cancer center. However, it remains completely unclear as to which of the required measures are truly desired by patients who undergo prostatectomy and to what extent these supportive measures are offered by urological clinics. Radical prostatectomies are in part performed outside of certified centers in Germany, so it is of interest to investigate to what extent these clinics also provide the supportive measures required by the certification catalog of the German Cancer Society. A study evaluating the offer of patient-desired supportive measures in the setting of radical prostatectomy has not yet been conducted, which means that the impact of these measures on patient satisfaction cannot currently be assessed. Furthermore, there are no comparative studies on certified and non-certified centers regarding the offer and the patients’ perception of these supportive measures. This gap is now being addressed by the results of our IMPROVE study, which encompasses 20 urological clinics.

It seems reasonable to assume that patients undergoing tumor surgery which is potentially associated with a relevant impact on functional outcome and the postoperative quality of life may benefit from having supportive measures offered to them before and after surgery. In their prospective, randomized study comprising 220 patients with colorectal carcinoma, Klinkhammer-Schalke et al. evaluated the impact of supportive measures (e.g., psycho-oncological support, social support, nutrition counselling, stoma care, fitness, physiotherapy) following colorectal surgery. For patients in the intervention group who were asked to select supportive measures according to their own perceived demand, they found a significantly higher quality of life 12 months after the surgical intervention [29]. Based on these results, it seems justified that the DKG defines supportive measures that have to be implemented prior to successful certification as a prostate cancer center. However, it seems astonishing that the different supportive measures have not been critically evaluated in terms of their relevance for individual patients with prostate cancer as long as 15 years after the initial establishment of the certification process for prostate cancer centers. 

Based on this first study investigating the impact of patients’ needs and the effective availability of supportive measures, the following important results from a patients’ perspective have to be highlighted:

- Patients have a very high need for pre-operative counselling concerning the best treatment option, which is not met sufficiently. Apparently, patients highly appreciate meeting their surgeon to answer any last questions prior to the surgical procedure. These two important points have to be considered even in economically driven healthcare systems that are associated with an ongoing increase in physicians’ workload.
- Contemporary studies have demonstrated that up to 31% of patients suffering from different levels of urinary incontinence which is defined as a need to be provided with at least one pad per day [30]. Therefore, it is understandable that patients show a high interest in perioperative supportive measures concerning pelvic floor exercises. In recent years, there is increasing evidence for additional pre-operative pelvic floor exercises as they are attributed to an increase in continence rates especially during the first months following radical prostatectomy [31]. Contrarily, our study reveals, as one of its most important findings, that patients are not counselled sufficiently about the potential impact of preoperative pelvic floor exercises.
- In our study, 25% of the entire cohort were aged 63 or younger at the time of radical prostatectomy. About 27% of them were professionally active at this time point. Hence, it is not surprising that postoperative rehabilitation addressing the recovery of physical fitness and sufficient social support were rated as highly relevant by the patients. Remarkably, no sufficient training offers and no adequate counselling concerning rational physical rehabilitation measures were offered to a substantial proportion of patients. In contrast, offering social support ensuring access to rehabilitation and improving communication with the employers of the patients seem to be better integrated into the daily routine of postoperative care.
- Surprisingly, our study found that perioperative offers concerning psycho-oncological support and the integration of patients with prostate cancer into a support group were not rated as very relevant by the patients. As there is a number of studies demonstrating the substantial need for such offers, it seems of utmost importance that these offers are explained to patients more intensively by their physicians [32,33].
- Interestingly, the offer of postoperative counselling regarding the therapy options for possible erectile dysfunction was not rated as very relevant by the patients, although the predefined definition for a very relevant supportive measure was only just missed. An impairment of erectile function, including clinically manifest erectile dysfunction, was reported in up to 88% of patients following radical prostatectomy, which assumes the high relevance of such supportive offers to patients. Again, this highlights the need for urologists to better communicate with their patients about existing supportive measures and their potential benefit to the patients [34].

Due to the retrospective nature of this study, our study has some limitations that have to be considered. First, our analysis may be prone to potential confounders. Although both groups in this study (CERTs and NCERTs) showed no essential differences in most of the demographic criteria, the patients were younger (67 vs. 69 years, *p* < 0.001) in CERTs. Regarding oncological criteria, a higher percentage of ISUP groups 3–5 (45.4% vs. 34.8%, *p* = 0.005) and a slightly higher rate of positive surgical margins (26.7% vs. 20.0%, *p* < 0.042) were found in CERTs as opposed to NCERTs. Of note, robot-assisted radical prostatectomy was performed more frequently in CERTs vs. NCERTs (68.3% vs. 14.8%, *p* < 0.001). Not surprisingly, in terms of the centers’ level of care, CERTs were statistically more often university centers (66.2% vs. 0%, *p* < 0.001) and had a higher annual caseload of radical prostatectomies (125 vs. 29, *p* < 0.001) compared to NCERTs. 

Variations in the time intervals between radical prostatectomy and assessments of the ratings of the different supportive measurements in the two groups, CERTs and NCERT (14 vs. 17 months, *p* < 0.001), may possibly have hampered the interpretation of our results, as an increasing decision regret over time was reported by Hurwitz et al. in patients who underwent radical prostatectomy [35]. This indicates that patients’ preferences and attitudes may vary over the course of time. However, as published previously, the time interval between radical prostatectomy and the survey was not significantly associated with patients’ decision regret within the IMPROVE study [20]. Therefore, although a statically significant difference in terms of this time interval was observed, it seems unclear how relevant this rather little variation might be. Nevertheless, the fact that patient-reported outcomes have been assessed only once after radical prostatectomy instead of assessed at various predefined points in time represents a limitation of this study.

The impairment of sexual function following radical prostatectomy was not evaluated in this study. As a consequence, the results might be biased, as patients may rate the relevance of supportive measurements differently depending on their individual impairment of sexual function (especially postoperative counselling regarding therapy options for possible erectile dysfunction). Different studies have shown the independent impact of sexual function on patients’ decision regret following radical prostatectomy for prostate cancer [36,37]. Unfortunately, no assessment of erectile function prior to surgery was available in a relevant number of participating centers. A retrospective assessment of preoperative erectile function would not have provided reliable data. No such evaluation was included in our questionnaire that was mailed to patients after radical prostatectomy, as only the impairment of sexual function opposed to the status before surgery would have represented a scientifically relevant criterion. Remarkably, the extent of intraoperative nerve sparing (no nerve sparing vs. unilateral or bilateral nerve sparing) showed no statistically significant differences between CERTs and NCERTs in this study. 

Finally, the fact that the definition of a supportive measure was arbitrarily considered as very relevant only if it had been rated as “very important” by >50% of patients and “rather important” or “very important” by >80% of patients represents another limitation of this study. However, no definition for the impact of a given supportive measure in the patients’ perspective has yet been established in the international literature. Therefore, no such definition could have been adopted as a standard.

To overcome the shortcomings associated with the retrospective nature of this study, it seems highly recommendable to initiate a prospective, randomized study comparing the perioperative standards of care in the context of radical prostatectomy with a tailored concept that incorporates the intensified supportive measures found to be particularly desired by patients based on the retrospective data highlighted in this study. For these two groups, patient-relevant endpoints, such as quality of life, decision regret, functional outcome and other patient-reported outcome measures, have to be evaluated at different time points postoperatively, similar to a study that has already been conducted for colorectal carcinoma [29].

Despite the limitations listed above, the IMPROVE study represents the first study worldwide to investigate a patient-reported assessment of the supportive measures offered to patients undergoing radical prostatectomy.

## 5. Conclusions

The IMPROVE study represents the first study worldwide to evaluate a patient-reported assessment of the supportive measures that accompany radical prostatectomy. The availability of pertinent offers varies marginally between CERTs and NCERTs, with no statistically significant difference observed concerning the supportive measures rated most relevant by the patients.

The identification of crucial supportive measures by the patients enables the intensification of such offers in the future, as some of them are currently estimated as insufficient and for some of them, a higher demand is postulated. On the other hand, some items have been attributed with a lower relevance by the patients in our study, although the impact of these measures on important outcome parameters has been shown before, indicating the need to council patients better about the potential benefit of these measures. Finally, as a consequence of this study’s results, a prospective, randomized study comparing the perioperative standards of care with a tailored concept incorporating intensified supportive measures is recommended in order to evaluate the impact of this approach on patient-relevant endpoints, such as quality of life and decision regret, at different time points postoperatively.

## Figures and Tables

**Table 1:** Descriptive characteristics of 750 patients who underwent radical prostatectomy for prostate cancer.

Variable	Entire Cohort (*n* = 750)	CERTs (*n* = 480)	NCERTs (*n* = 270)	*p* Value
Age (*n* = 750):				<0.001
median (IQR) in years	68 (63–72)	67 (62–71)	69 (64–73)	
Personal relationship status (*n* = 747):				0.071
fixed partnership	675 (90.4%)	440 (91.9%)	235 (87.7%)	
no fixed partnership	72 (9.6%)	39 (8.1%)	33 (12.3%)	
Social security status (*n* = 750):				0.864
statutory health insurance	548 (73.1%)	352 (73.3%)	196 (72.6%)	
private health insurance	202 (26.9%)	128 (26.7%)	74 (27.4%)	
Educational qualification (*n* = 747):				0.566
university or technical college degree	237 (31.7%)	156 (32.6%)	81 (30.2%)	
no such qualification	510 (68.3%)	323 (67.4%)	187 (69.8%)	
Professional status (*n* = 745):				0.069
professionally active or professional activity scheduled again	198 (26.6%)	138 (28.9%)	60 (22.5%)	
retired	547 (73.4%)	340 (71.1%)	207 (77.5%)	
Time interval between RP and survey in month (IQR) (*n* = 750)	15 (11–21)	14 (11–21)	17 (12–21)	<0.001
Clinical decision making regarding surgical approach (*n* = 742):				0.051
Decision by physician alone (passive decision)	181 (24.4%)	105 (22.0%)	76 (28.7%)	
Consensual				
(patient and physician together)	361 (48.6%)	232 (48.6%)	129 (48.7%)	
Decision by patient alone (active decision)	200 (27.0%)	140 (29.4%)	60 (22.6%)	
Center’s level of care:				<0.001
non-university center	432 (57.6%)	162 (33.8%)	270 (100%)	
university (*n* = 750)	318 (42.4%)	318 (66.2%)	0	
Center’s mean RP caseload per year 2018–2020 (IQR) (*n* = 750)	87 (52–134)	125 (67–150)	29 (19–92)	<0.001
Preoperative PSA level in ng/mL (IQR) (*n* = 703)	7.9 (5.6–12.1)	7.7 (5.4–12.3)	8.4 (6.0–12.0)	0.224
ISUP group 1–2	438 (58.4%)	262 5(54.6%)	176 (65.2%)	0.005
(Gleason score 3 + 3 = 6 and 3 + 4 = 7)				
ISUP group 3–5	312 (41.6%)	218 (45.4%)	94 (34.8%)	
(Gleason score 4 + 3 = 7, 4 + 4 = 8, 3 + 5 = 8, 5 + 3 = 8, 4 + 5 = 9, 5 + 4 = 9, and 5 + 5 = 10)				
(*n* = 750)				
pT stage (*n* = 750):				0.057
pT2	482 (64.3%)	296 (61.7%)	186 (68.9%)	
pT3 + pT4	268 (35.7%)	184 (38.3%)	84 (31.1%)	
pN stage (*n* = 749):				0.283
pN0 + pNx	683 (91.2%)	441 (92.1%)	242 (89.6%)	
pN1	66 (8.8%)	38 (7.9%)	28 (10.4%)	
Surgical margin status (*n* = 750):				0.042
R0	568 (75.7%)	352 (73.3%)	216 (80.0%)	
R1	182 (24.3%)	128 (26.7%)	54 (20.0%)	
no adjuvant local radiation adjuvant local radiation (*n* = 746)	614 (82.3%) 132 (17.7%)	389 (81.2%) 90 (18.8%)	225 (84.3%) 42 (15.7%)	0.318
Nerve sparing (*n* = 703):				0.074
no nerve sparing	275 (39.1%)	200 (41.7%)	75 (33.6%)	
unilateral nerve sparing	108 (15.4%)	75 (15.6%)	33 (14.8%)	
bilateral nerve sparing	320 (45.5%)	205 (42.7%)	115 (51.6%)	
Postoperative complications according to CDC grades (*n* = 703):				0.087
0–2	662 (94.2%)	447 (93.1%)	215 (96.4%)	
3–5	41 (5.8%)	33 (6.9%)	8 (3.6%)	
Urinary stress incontinence (*n* = 747):				0.131
0–1 safety pad/day	594 (79.5%)	389 (81.2%)	205 (76.5%)	
>1 pad/day	153 (20.5%)	90 (18.8%)	63 (23.5%)	
Surgical approach (*n* = 750):				<0.001
open surgical procedures	325 (43.3%)	110 (22.9%)	215 (79.6%)	
laparoscopic (not robot-	57 (7.6%)	42 (8.8%)	15 (5.6%)	
assisted)				
robot-assisted procedures	368 (49.1%)	328 (68.3%)	40 (14.8%)	

Legend: CDC, Clavien–Dindo classification; CERT, certified prostate cancer center; IQR, interquartile range; ISUP, International Society of Urological Pathology; NCERT, non-certified center; PSA, prostate-specific antigen; RP, radical prostatectomy.

## Data Availability

The data presented in this study are available on request from the corresponding author.

## Collaborators

Patricia Lindberg-Scharf, Monika Klinkhammer-Schalke, Martin Baunacke, Marcus Sondermann, Johannes Huber, Rolf Gillitzer, Julie Steinestel, Paola Schifano, Christian Bolenz, Sascha Pahernik, Clemens Huettenbrink, Maximilian Janssen, David Schilling, Bara Barakat, Christian Fuhrmann, Andreas Manseck, Robert Wagenhoffer, Wolfgang Schafhauser, Alexander Kugler, Ekkehard Geist, Lisa Blair, Jesco Pfitzenmaier, Bettina Reinhardt, Johannes Bründl, Philipp Karschuck, Amr Gaber Mahmoud, Tobias Jordan, Salah Al-Dumaini, Islam Yassin-Kassab, Raik Wustmann, Thomas Enzmann.
